# Assessing frailty in aged zebrafish using a quick pseudo-frailty index

**DOI:** 10.1242/bio.062345

**Published:** 2026-04-24

**Authors:** Anne Cathrine Hyde, Pamela Ellis, Sherif El-Khamisy, Claire Allen, Catarina M. Henriques, Fredericus J. van Eeden

**Affiliations:** ^1^ The University of Sheffield, School of Bioscience, Bateson Centre, Healthy Lifespan Institute Sheffield, Sheffield S10 2TN, UK; ^2^ The University of Sheffield, School of Medicine and Population Health, Bateson Centre, Healthy Lifespan Institute, Sheffield S10 2TN, UK; ^3^ Institute of Cancer Therapeutics, Faculty of Life Sciences, University of Bradford, Bradford BD7 1DP, UK; ^4^ The University of Sheffield, Biological Services Aquarium, Sheffield S10 2TN, UK

**Keywords:** Frailty, Frailty index, Zebrafish ageing, Zebrafish, Ageing

## Abstract

While biomedical advancements have significantly extended human longevity, increased lifespan is frequently decoupled from healthspan, as these additional years are often accompanied by frailty and chronic morbidity. Frailty is characterised by a multisystem decline in physiological reserve and renders individuals disproportionately vulnerable to adverse outcomes – including mortality – when faced with health stressors. Consequently, research into the biological mechanisms linking ageing to the onset of frailty is needed. The zebrafish is increasingly utilised as a model for human aging and frailty due to its high degree of genetic homology. With a short lifespan of approximately 3 years, zebrafish show several conserved senescent phenotypes, including cataracts, sarcopenia, spinal curvature, and motor decline. Crucially, as in humans, ageing in zebrafish is not strictly chronological; apparent biological age often diverges from calendar age, with physical condition indicating frailty status more accurately. We propose a frailty index designed to evaluate the divergence between successful ageing and frailty in zebrafish. By utilising easily quantifiable phenotypic markers such as spinal curvature and body mass index, this index provides a score that predicts frailty status as validated against expert assessment. Implementing this standardised metric will facilitate cross-laboratory comparisons and enhance the reproducibility of future zebrafish-based ageing research.

## Teaser

**Summary:** We present a simple scoring system for frailty zebrafish; it was optimised to best match scores from experts and allows standardisation of frailty measurements in fish ageing research across laboratories.

## INTRODUCTION

The definition of what conditions make a person frail has long been a debate, whether it means that a person should have an age when frailty can occur or that the person is reliant on services for help*.* Here, we refer to frailty as a change in physiological health that increases the chance of adverse outcomes after disease (Kane and Howlett, 2021; Rockwood et al., 1994, 2000). What is clear, however, is that people with the same chronological age can vary greatly in frailty, and that chronological age may not reflect biological ageing (Franceschi et al., 2018; Mitnitski et al., 2002, 2001). Therefore, when studying biological ageing, a consistent way to identify frail individuals is needed. This can be provided by a frailty index or a frailty phenotype, which identifies deficits in health, and the more deficits an individual accumulate, the greater frailty they have (Heinze-Milne et al., 2019; Rockwood and Mitnitski, 2011; Searle et al., 2008). One widely used score in humans is the Fried frailty score. It assesses unexpected weight loss, poor handgrip, slow speed gait, feelings of exhaustion and low physical activity. If an individual, usually over 65 years of age, scores on three or more of the five criteria, they are classed as frail and have an increased risk of disease, and death within the next 3 years (Franceschi et al., 2018; Fried et al., 2001). Frailty scores have been created for model organisms. In mice, frailty is usually measured by activity levels, body composition, heart conditions, and metabolic status. However, a non-invasive measurement with eight conditions has also been shown to predict frailty (Parks et al., 2012; Rockwood et al., 2017; Whitehead et al., 2014).

Zebrafish ageing has several features that resemble human ageing and are increasingly adopted as models to study the ageing process due to their short lifespan and low cost (Gilbert et al., 2014; Khor et al., 2019; Abou-Dahech and Williams, 2024; Hudock and Kenney, 2024). However, like in humans, zebrafish chronological age does not reflect their biological age, and there is no one way to determine what fish is frail and has an increased chance of death when faced with adversity. Several physiological features have been associated with increasing age in zebrafish: gross morphological changes in spinal curvature and loss of muscle mass, with severity increasing over time (Gerhard and Cheng, 2002; Hayes et al., 2013). Further, zebrafish have an ability to regenerate damaged tissue; however, this ability decreases with age (Gilbert et al., 2014). An increase in cancer occurrence is also associated with increasing age in zebrafish (Cayuela et al., 2018; Kishi et al., 2003; Laconi et al., 2020). Other features that also change with zebrafish age include cataract, reduced swimming speed, cognitive decline and cardiovascular dysfunction (Gilbert et al., 2014; Khor et al., 2019).

Flueck-Giraud et al. (2024) recently published a score sheet to assess the welfare of experimental fish. There they assess several external signs of welfare such as body condition as well as behavioural signs (Flueck-Giraud et al., 2024). However, assessing general welfare of the animal may tell you more about the state of the fish at that moment, rather than their frailty status linked to ageing. The Henriques laboratory published promising evidence for using width/length ratios as a way to assess frailty as a frailty index. They have shown that in prematurely ageing *tert*^−/−^ fish there is a decrease in the width and length ratio of the fish (Ellis et al., 2024; Henriques et al., 2013). However, it is unclear whether there might be other markers in fish that can be used to optimise the assessment of frailty.

In this study, we aimed to create a quick way of assessing the frailty status of zebrafish starting with only five conditions. We took inspiration from other organisms and humans, and selected markers for frailty that include body mass index (BMI), which changes in humans with age, with greater weight loss in frail humans (Crow et al., 2020), spinal height, presence of tumours and the presence of fin degeneration. Frailty is difficult to define and is mainly interpreted as an increased chance of dying after an insult; however, ethical considerations make it impossible to verify the ‘correctness’ of our score. As an alternative, we asked zebrafish experts to score fish on frailty and modified the relative weights of our factors to get the optimal match to their average scores. We propose that either the original or the optimised score can be used to allow standardisation of frailty measures across different laboratories.

## RESULTS

### Frailty phenotypes increase with age

**Fig. 1.**
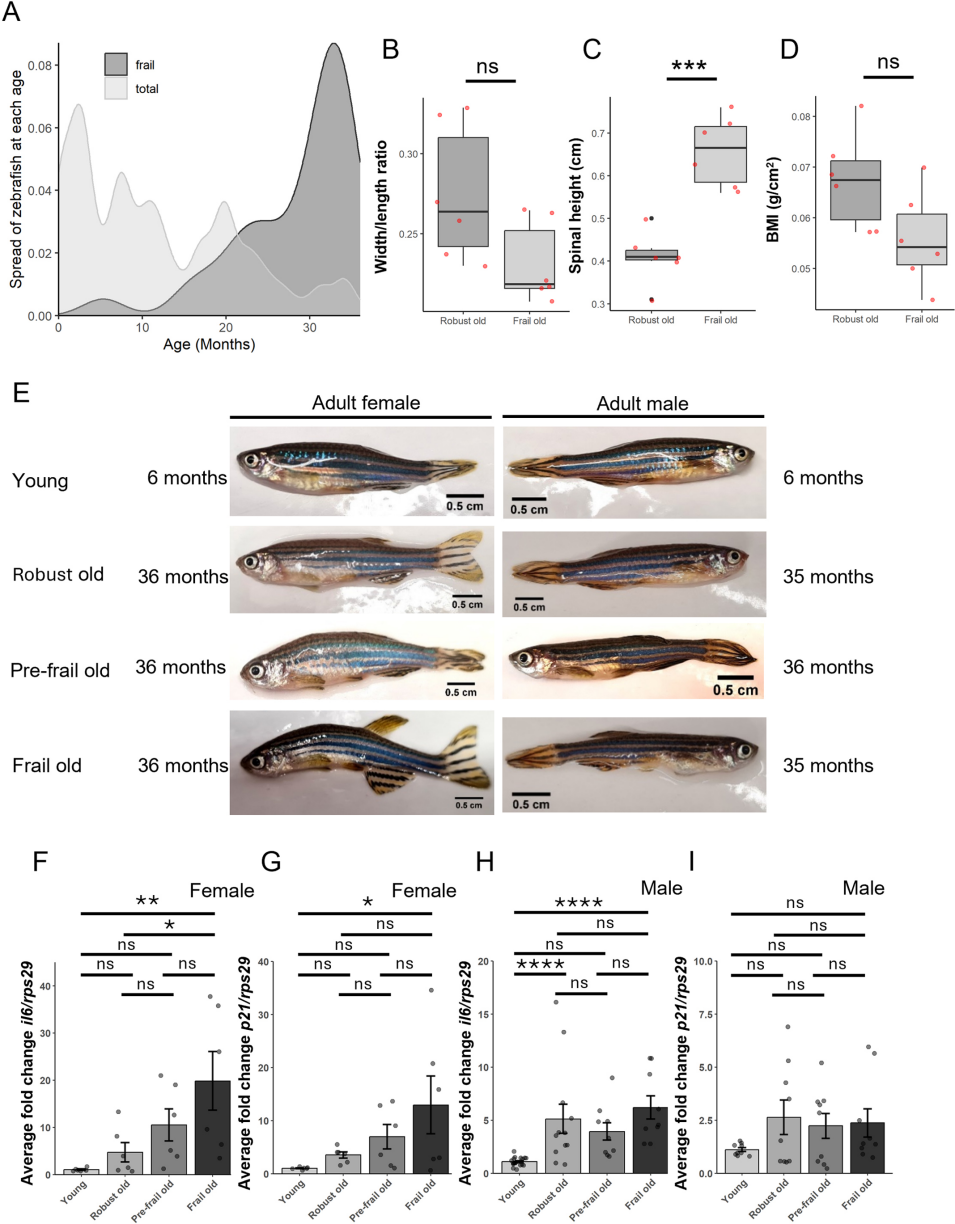
**Creating the pseudo-frailty index for ageing zebrafish.** (A) Density plot showing the ages of the total number of fish in the aquarium (light grey) against the spread of the ages the fish in the aquarium can be classed as frail based on the frailty index (dark grey). (B-D) Initial width/length ratio (A), BMI (g/cm^2^; B) and spinal height (cm; C) values from six visually robust and six visually frail female fish that created the frailty index. Unpaired *t*-test, *n*=6. (E) Images of female and male zebrafish in the four different frailty groups: robust young, robust old, pre-frail old, frail old. The female robust old and frail old fish are siblings, as are the males. (F) qPCR of female tail fins for *il-6*, *n*=6. (G) qPCR of female tail fins for *p21*, *n*=6. (H) qPCR of male tail fins for *il-6*, old *n*=9, pre-frail *n*=9, robust *n*=15, young *n*=18. (I) qPCR of male tail fins for p21, old *n*=9, pre-frail *n*=9, robust *n*=9, young *n*=9. ANOVA (ns, not significant; *P<0.05, **P<0.01, ***P<0.001, ****P<0.0001).

We initially explored if the aforementioned five frailty phenotypes did increase in a population of fish with age. The age of the fish in the aquarium that visually had any of the five criteria were noted and plotted against the age of the total number of fish in the aquarium. This density plot (Fig. 1A) shows that the number of fish that can be classed as frail according to our frailty system increases with age, peaking at 33 months.

### Creating cut-offs for the frailty index

To create a preliminary zebrafish frailty index, we measured six robust and six frail female fish that were identified as robust or frail visually according to the five frailty criteria. We then created cut-offs to distinguish between robust and frail values (Fig. 1B-D). By using the cut-offs identified by these (Table 1), we scored subsequent fish and four groups of adult fish were created, robust young (3-6 months; score 0), robust old (3 years; score 0), pre-frail old (3 years; score 0.2) and frail old (3 years; score 0.4+) (Fig. 1E). The male cut offs were created in a similar fashion to the female cut offs. We also performed quantitative PCRs (qPCRs) on RNA from fin clips of two frailty-associated genes, *il-6* and *p21* (Fig. 1F-I). We used *ribosomal protein 29* (*rps29*) as our housekeeping gene as it is commonly used in zebrafish research (Bower et al., 2017; Fargher et al., 2025; Coxam et al., 2014; Kim et al., 2020).

**Table 1.** Scoring system for pseudo-frailty scale for zebrafish

Criteria	Female threshold	Male threshold	Scoring
Width/length ratio	0.23	0.22	<=1>=0
BMI (g/cm^2^)	0.056	0.041	<=1>=0
Spinal curvature	0.55	0.4	>=1<=0
Fin degeneration	Yes/No	Yes/No	Yes=1, No=0
Tumours	Yes/No	Yes/No	Yes=1, No=0

### Scoring of old fish from proposed criteria limits

Using the criteria from Table 1, females and males scored differently at the different criteria (Table 2). It was more difficult to score males as their characteristics are less obvious. Whereas the females scored on criteria that are very visual, such as the decreased BMI and increase in spinal curvatures, males mostly scored on a decrease in width/length ratio with a decrease in BMI in second place.

**Table 2.** Percentages of total and frail-scoring female and male that scored on each criterion

Criteria	Female		Male	
	Total (*n*=69)	Frail (34.5%)	Total (*n*=44)	Frail (25%)
Width/length ratio	18.8%	52%	36.4%	72.7%
BMI (g/cm^2^)	42%	96%	15.9%	54.5%
Spinal curvature	39.2%	79%	18.2%	45.5%
Fin degeneration	8.7%	20%	9.1%	18.2%
Tumours	7.2%	12%	11.4%	36.4%

### Frailty scoring by zebrafish experts

**Fig. 2.**
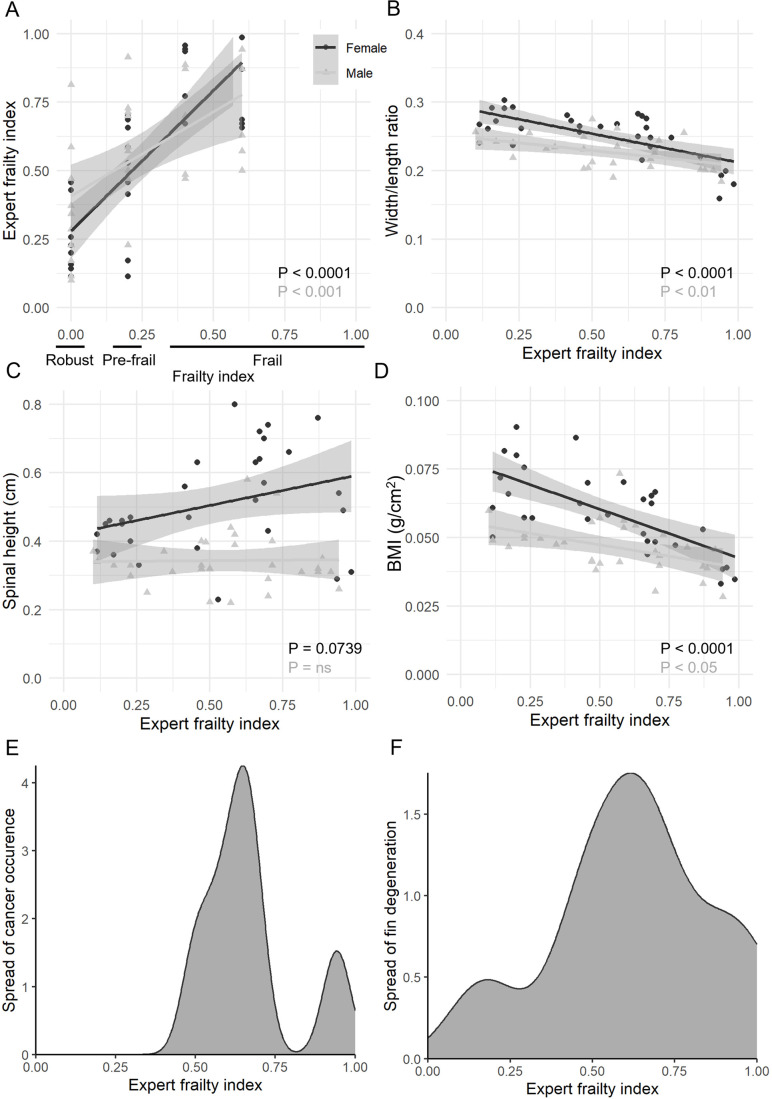
**Indexed frailty scoring of young, old robust, pre-frail and frail old female and male zebrafish by zebrafish experts.** (A) The average score per fish by the experts increases significantly with the frailty index. (B) The width/length ratio decreases significantly with higher scores given by the experts. (C) No significant increase in spinal height with higher scores given by experts. (D) The fish BMI decreases significantly the higher the score the experts gave the fish. A, *n*=40; B-D, *n*=30. Simple linear regression. (E) Density plot of tumour occurrence amongst the 60 old fish against the experts' scores. *n*=6. (F) Density plot of fin degeneration amongst the 60 old fish against the experts' scores. *n*=8.

To validate our frailty index, we had seven zebrafish experts, each with 15 years or more experience in using and caring for zebrafish, score images of 80 fish (ten each from our categories young, robust old, pre-frail old, and frail old; both sexes). For both females and males, the expert scores generally increased with each frailty classification (Fig. 2A). However, there were some variations among the scores. Female fish that were thin uniformly scored highly, whilst fish with spinal curvatures that had varying degree of width had a variety of scores from 0.2 to 1. Regression confirmed that BMI and width/length ratio significantly decrease with higher frailty; however, the spinal height does not (Fig. 2B-D). For the males, again, thin fish uniformly scored high; however, fish that were not obviously thin by eye were given scores between 0.6 and 1, meaning that male fish that may have only scored on the width/length ratio and be classed as pre-frail in the scoring system might visually be frail even though they had an average BMI. Further, when plotting the density for tumours and fin degeneration against the expert scores, we saw that both criteria were mostly seen in fish that scored higher on the frailty score (Fig. 2E,F). Overall, this suggests that phenotypic scoring of frailty appears more straightforward for females than for males.

### Optimising frailty index with weighted criteria

**Fig. 3.**
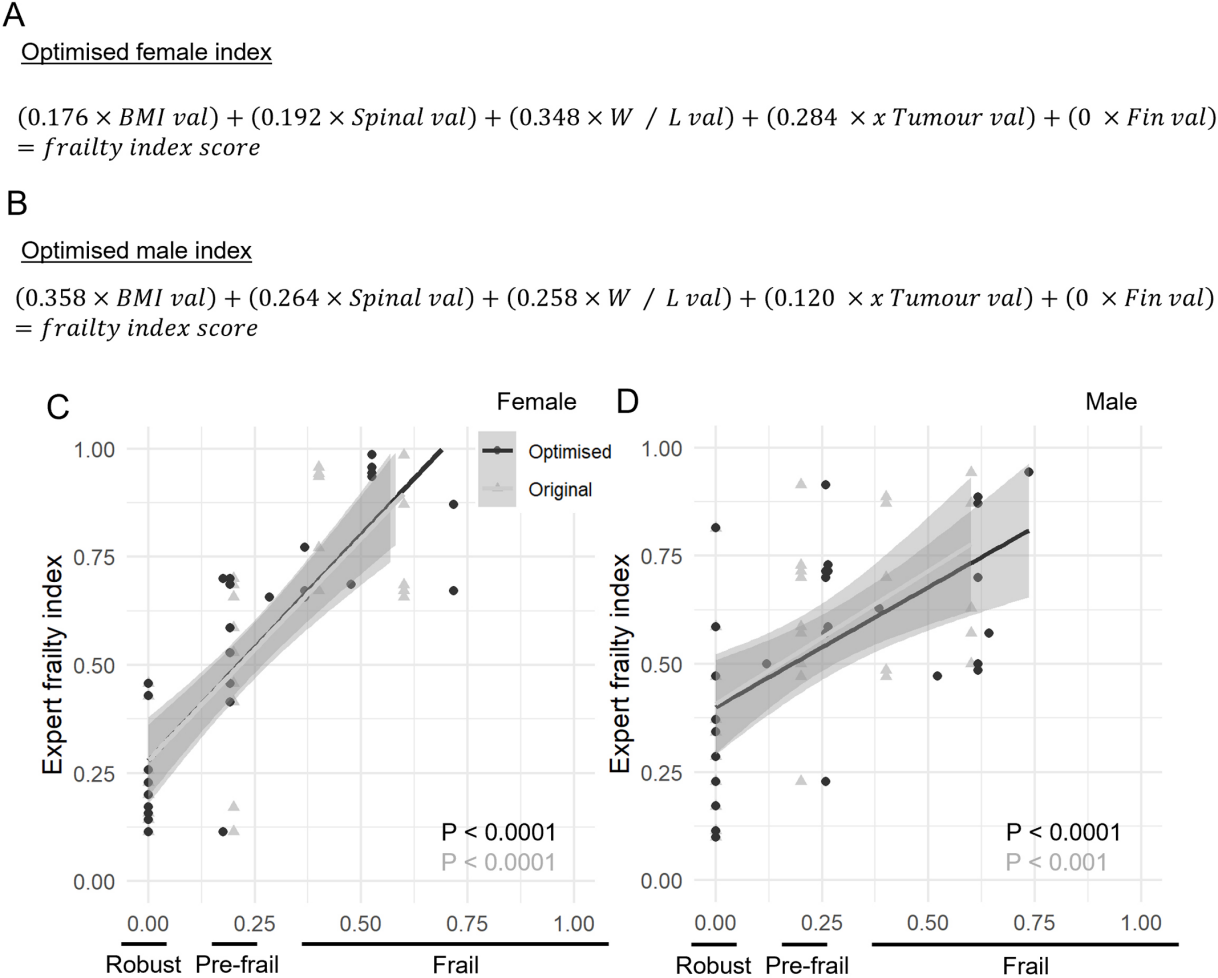
**Optimisation of the zebrafish frailty index.** (A,B) Female (A) and male (B) equations for optimised frailty index with weights based on expert scores. The values (val) relate to the 1 or 0 given to the fish from Table 1, as to whether they have the frailty feature or not. (C,D) Frailty scoring of 60 old robust, pre-frail and frail old female (C) and male (D) zebrafish by zebrafish experts compared to the optimised frailty index with weighting and the original frailty index proposed in Table 2. Simple linear regression, *n*=30.

We used linear regression to refine the frailty score, aiming to align it more closely with the judgment of our experts. The expert scores were used as a dependent variable whereas the scores of each individual criterion (Table 1) acted as the independent variables. The resulting slope coefficients were used to create weights, which were then normalised to get a total score of 1 if a fish scored on all criteria. For the female and the male fish, the weight for the fin degradation were negative, suggesting that the experts considered it unimportant; therefore, this criterion was effectively removed from the score as the weight was set to 0. The equations for the optimised index are shown in Fig. 3A,B and confirm strong correlation with expert scores.

We used root mean square error (RMSE) to confirm that our new score was a better fit for the expert data. Using the indexed expert scores as the predicted values, we calculated the RMSE against two sets of actual values: the original total score and our newly optimised total score. The original RMSEs were 0.335 for females and 0.385 for males, improving to 0.326 for females and 0.358 for males after weighting. When only looking at the width and length ratios as used by the Henriques laboratory, again using the indexed expert scores as the benchmark and the width/length ratio as the actual score, the RMSE for the females was 0.405 and for the males 0.410.

## DISCUSSION

Ageing research is becoming increasingly important, but aged animals are difficult to obtain. Large zebrafish facilities often generate such fish as part of standard husbandry procedures, and these could be a valuable resource to study ageing. Like humans, zebrafish appear to age differentially, with some becoming frail earlier than others. In order to classify such animals consistently we propose a quick pseudo-frailty index to allow consistent scoring of fish. We use the term ‘pseudo-index’ because it has not used death as the frailty outcome, due to ethical considerations and UK legislation.

Initially, we used five criteria, BMI, width/length ratio, spinal curvature, tumours and fin degeneration. Fish that score on none of the criteria are classed as robust, one criterion are classed as pre-frail and two or more we class as frail fish. To make the index more accurate, we used linear regression analysis to assign different weights to criteria, trying to best match the expert scores. Due to physiological differences between the female and male fish, we created sex-specific thresholds and weights.

Our males scored less on the unmodified frailty index, possibly due to bias; we have found that in older stocks females appear overrepresented, suggesting that males may have to be removed earlier for health reasons. Controlled raising experiments would be required to verify this. Similar trends occur in the human population, where females tend to become frailer than men, even though they also live longer (Kane and Howlett, 2021; Park and Ko, 2021). Female wild-type mice reportedly have an increased frailty index (Whitehead et al., 2014); however, other studies contest this (Parks et al., 2012). Furthermore, more frail aged guide dogs were female (Hua et al., 2016).

While all organisms experience frailty uniquely, we observed significant intra-sex variation but also clearly distinct patterns of frailty between male and female zebrafish. We generally see that females most often scored based on a BMI, likely because they lose eggs as they age, causing them to thin. For males, the dominant criterion is the width/length ratio. Tumours are seen frequently in both sexes, and have a prevalence of increasing with age (Laconi et al., 2020). Further, tumours in zebrafish can be fairly easy to recognise and use as a quick score. An argument for adding fin defects is that this may point to defective fin regeneration (Pfefferli and Jazwinska, 2015), which is a sign of ageing (Gilbert et al., 2014; Kishi, 2004). Interestingly, the loss of fin regeneration was not seen as important to score frailty by the experts, and this criterion was therefore removed from the optimised index.

We considered and ultimately excluded several other criteria for our index. Cataracts, for example, are associated with age and frailty (Mencucci et al., 2023). However, robustly identifying cataract in aged zebrafish was too difficult to do reliably. Similarly, skin ulcers or scale damage was considered, but these were rarely observed in the aged fish and can also occur in young adults. They could be a general welfare marker rather than an ageing feature (Flueck-Giraud et al., 2024). We chose not to include reduced movement despite linkage to frailty and use in several frailty scoring systems (Billot et al., 2020; Franceschi et al., 2018; Fried et al., 2001); the need for specialised equipment and time needed made this less desirable. Width-length ratio and spinal curvature may indirectly reflect movement as it will be affected by loss of muscle mass (Gilbert et al., 2014). We also chose to exclude genetic and molecular markers from the index as we wanted to create a rapid index that required little equipment and that would create a general index that was not limited towards one aspect of ageing, such as tying the index down to inflammation or nutrient sensing. Of course, we hope that future experiments could now use this index to examine correlations between the index and molecular and behavioural parameters.

We analysed the expression of two established frailty markers, *il-6* and *p21*, in combination with our frailty index (Fig. 1F-H) (Marcozzi et al., 2023; Puts et al., 2005). An orthologue of *il-6* has been identified in zebrafish and has previously been reported in the adult caudal fin after injury. An increase in *il-6* has also been observed in *sert1^−/−^* prematurely ageing zebrafish larvae and internal organs (Bohaud et al., 2021; Mastrogiovanni et al., 2024; Abou-Dahech and Williams, 2024; Kim et al., 2019). Expression of *p21* has also been observed in zebrafish larval fins after amputation, and is recognised as a key senescence and ageing marker in zebrafish (Morsli et al., 2023; Da Silva-Álvarez et al., 2020). We found that in female fish, the expression of both markers steadily increases with frailty. Whilst there is a significant change in *il-6* between robust and frail old fish in females, there is only a trend for this observation for *p21.* This could suggest that for frailty, inflammation is a more sensitive marker than senescence. Further, for the male fish, we only saw significant changes between young and old fish, not between the old groups. This could be a result of identifying the three different classes of old fish being more difficult in males than females. The results from the qPCR, therefore, might suggest that the frailty index works well to separate out the different frailty groups of female zebrafish; but, it might not be as sensitive for male zebrafish. Alternatively, it has also been seen in mice that females tend to show a stronger correlation between inflammation and frailty than males (Kane et al., 2019), which could also be a reason why the markers are not significant in male fish, and perhaps other frailty markers such as endocrine markers could be used to further assess the molecular patterns within the frailty groups.

To validate our frailty index further, we had seven zebrafish experts visually assess and score 80 fish, which included young, robust, pre-frail, and frail individuals. The experts' scores showed a strong positive correlation with our frailty index (Fig. 2A). We noted that some criteria seem less important for frailty. Both, BMI and width/length ratio showed significant negative regression with increasing frailty scores (Fig. 2B,C); however, this was not the case for the spinal curvature as it was not significant with increasing frailty, although there was a trend with the females but not the males (Fig. 2D). Additionally, whilst tumours and fin degeneration were not as important for high frailty, it appeared that fish with either of these criteria scored pre-frail or higher (Fig. 2E,F).

By using linear regression, we optimised our frailty index to better reflect expert judgments. The resulting slope coefficients were used as normalised weights, as seen in Fig. 3A,B, to determine the importance of each criterion. This process revealed that fin degeneration was not a significant marker of frailty and was therefore removed from the index. Interestingly, the width-to-length ratio proved to be the most critical criteria for the females whilst BMI was the most critical for males. This observation is opposed to the percentages calculated for which observations were more common in each sex seen in Table 2. Applying this new, four-parameter index to the 60 fish scored by experts, we found a stronger correlation with the expert scores than with the original index (Fig. 3C,D). An RMSE analysis confirmed that our optimised system provided a more accurate assessment of frailty. Additionally, our optimised index performed better than a previously used method that relies solely on the width/length ratio to estimate frailty (Ellis et al., 2024; Henriques et al., 2013), as indicated by a less favourable RMSE for that approach.

This study introduces an optimised, four-criteria index designed to quantify frailty in zebrafish through easily accessible parameters. We acknowledge that this index is currently calibrated against subjective expert assessment rather than longitudinal survival data, which remains a limitation. Furthermore, as our subjects were sourced from diverse genetic backgrounds, potential heterozygosity may have exerted subtle influences on morphology. Nevertheless, this index provides a vital framework for the standardisation of frailty metrics across laboratories. It serves as a valuable tool for distinguishing between robust and frail aging trajectories and offers a practical application for monitoring animal welfare within aquarium facilities

## MATERIALS AND METHODS

### Zebrafish husbandry

Zebrafish were maintained at the Bateson Centre aquarium at the University of Sheffield under site licence X57506C3D, complying with UK laws. They were kept in freshwater at 28°C with a 14:10 h light:dark cycle at a density of five animals per 1 l or less. They received either dry food (ZEBRAFEED, Sparos lda) or live artemia according to Dataset 1. A health screen report and water quality parameters are also available in Dataset 1. No fish were bred for the purpose of this study; only surplus fish from the aquarium were used to make this scoring system. Animals may have had wild-type or heterozygous backgrounds that do not produce an adult phenotype (Dataset 2).

### Culling and measurements of zebrafish

**Fig. 4.**
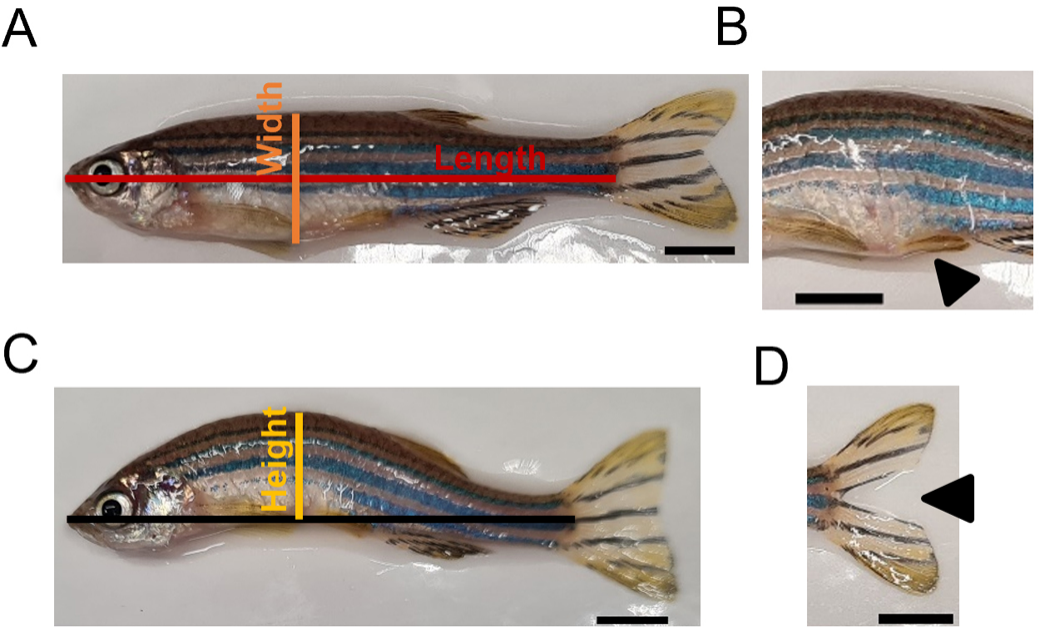
**Examples of how measurements were taken for the frailty index.** (A) Length (red) was measured from tip of the nose to the start of the fin in a straight line. Width (orange) was measured from the top of the spine to the bottom of the stomach in a straight line. (B) Example of a tumour in the stomach region. (C) The spinal height (yellow) was measured from the line used to measure length to the top of the spine. (D) Example of a split fin. Arrowheads indicate tumor (B) or fin defect (D). Scale bars: 0.5 cm.

Adult zebrafish (aged 4-9 months and 35-36 months for females, 4-9 months and 23-36 months for males) were culled using tricaine (MS-222, Bioserv UK Ltd) followed by destruction of the brain (UK schedule 1 procedure) and imaged on a scale with a ruler. Their weight, sex, tumours, and frayed fins were recorded. The tail fin was clipped for RNA extraction. Images were analysed using Fiji/ImageJ. Fish length (cm) was measured from the tip of the mouth, in a straight line to the start of the tail fin (Fig. 4A). The height of the spinal curve was measured from the top of the spine to a line drawn from the mouth of the fish to the middle of the fin (Fig. 4C). The fish width was measured from the top of the spine to the bottom of the stomach (Fig. 4A). A width-to-length ratio was calculated, and the BMI of the fish was determined by taking the weight in grams of the fish and dividing it by the square root of the length of the fish. Tumours were identified as lumps in the fish (Fig. 4B), while fin degeneration was identified by split or frayed fins (Fig. 4D). We followed existing scoring systems such as the Fried index, which uses a step function (Fried et al., 2001) rather than a continuous scoring system.

### qPCR

The qPCR experiments followed the Minimum Information for Publication of Quantitative Real-Time PCR Experiments (MIQE) standards presented in Bustin et al. (2009). Tail fin clips from culled adult young, robust, pre-frail and frail old fish were snap frozen on dry ice, then either stored at −70°C or processed immediately by crushing the fin clip using a micropestle rotor, and RNA was extracted using Trizol and chloroform. RNA concentration and purity was measured using a nanodrop (ND-100 v3.8.1, Labtech), and stored at −70°C. Young fish were used as the control group. 470 ng/µl RNA was converted to cDNA using LunarScript RT SuperMix (M3010L, NEB) kit in a 10 µl reaction following the kit instructions. cDNA was stored at −20*°C.* qPCRs performed with HOT FIREPol EvaGreen qPCR Mix Plus (no Rox) (Solis Biodyne) in a 20 µl reaction with 0.5 µM of primers [*il-6* Fw: 5′TCAACTTCTCCAGCGTGATG3′ Rev: 5′TCTTTCCCTCTTTTCCTCCTG3′ (Safari et al., 2016), *p21* Fw: 5′AGGAAAAGCAGCAGAAACG3′ Rev: 5′TGTTGGTCTGTTTGCGCTT3′ (Morsli et al., 2023) and *rps29* Fw: 5′TTTGCTCAAACCGTCACGGA3′ Rev: 5′ACTCGTTTAATCCAGCTTGACG3′ (Bower et al., 2017)] in a Bio-Rad (HSP9601) 96-well qPCR plate. The following cycling conditions were used with wild-type cDNA on 0, −1, −2, −3 times dilutions to confirm that the primers had an amplification efficiency of between 90% and 110% and, in the experiments with cDNA diluted −1, 95°C 10 min, then 45 cycles of 95°C 15 s, 55°C 15 s, 72°C 30 s. The fluorescent intensity in the reaction was read at the end of each cycle. The amplification efficiently was calculated using the equation for primer efficiency in Bio-Rad's Application Guide for Real-Time qPCR (2006):

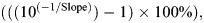
where the slope was calculated from the average Ct value from the three replicates of the diluted wild-type cDNA, which was used to construct a standard curve by plotting the log of the starting quantity of the template (0, −1, −2, −3) against the average Ct. The specificity of the primers was further evaluated by looking at their melting curve of the amplified product. The threshold for Cq value was set automatically by the Bio-Rad (CFX96^TM^ Real-Time System) as the beginning of the exponential phase during the 45 cycles, and the Cq value was identified by the machine when the fluorescent intensity within a sample reached that threshold. Three replicates of each sample were run, as well as three replicates of water that were used as contamination control for each primer set for every qPCR. A qPCR run was deemed not contaminated if the Cq value of the water samples were not amplified or higher than 45 cycles. The Ct values for the samples were calculated by subtracting the Cq value of *rps29* (housekeeping) from the Cq values of each corresponding target gene for each replicate creating a ΔCt value. A ΔΔCt value was calculated by subtracting the average young ΔCt value for the gene from the ΔCt of the individual replicates. A fold change value for each replicate was calculated by taking the 2^-ΔΔCt^. The average fold change which was the plotted value was calculated by averaging the fold change value for the three replicates. Statistical analysis was performed in R and R-studio version 4.1.1 using one-way ANOVA with a TukeyHSD (Honesty Significant Difference) post-hoc test.

### Frailty scoring by zebrafish experts

Ten images each of culled female and male young, robust old, pre-frail old and frail old fish (total=80) (Dataset 4) as classed by the proposed system were shown to experienced Named Aquarium Care and Welfare Officer (NACWO)-trained aquarium staff and other zebrafish ageing researchers (*n*=7); all have more than 10 years of experience caring and recognising old and frail fish, and are trained by using various body scoring criteria (Clark et al., 2018). They scored these by eye from 0 to 5, where 0 is robust and 5 is the frailest/sickest fish they have seen. The scores for each fish were averaged and indexed and plotted within each group. The raw expert data can be found in Dataset 3.

### Linear regression

To identify optimal weights for our criteria, we focused on the old fish, and did linear regression where the fish experts indexed values were used as the dependent variable, and each five criteria indexed were used as the independent variables. For the criteria, this means that if one of the old fish scored according to the threshold, that criterion would have the value of 0.2 for that individual fish, or 0 when negative. The slope coefficients obtained from the linear regression were used as the optimal weights. If any of the slope coefficients were negative, their weight was set to 0 as it would indicate that this criterion was not important for the experts. As the index needed to be a total value from 0 to 1, the weights were normalised so that if a fish scored on all the criteria, they would score 1 by dividing the weights by the sum of all the weights. For R code, see Dataset 5.

### Use of AI

AI was used to advise on efficient use of English in order to hit word limits for abstracts. No generative AI was used.

## Supplementary Material

10.1242/biolopen.062345_sup1 Supplementary information

Dataset 1.

Dataset 2.

Dataset 3.
