# Supplementary material for: Assessing frailty in aged zebrafish using a quick pseudo-frailty index
Source: Biol Open. 2026 Apr 24;15(4):bio062345. doi: 10.1242/bio.062345 (PMC13148474; doi:10.1242/bio.062345)
Supplement: Supplementary information [file biolopen-15-062345-s1.pdf]

Dataset 1.

Available for download at
https://journals.biologists.com/bio/article-lookup/doi/10.1242/bio.062345#supplementary-data

Dataset 2.

Available for download at
https://journals.biologists.com/bio/article-lookup/doi/10.1242/bio.062345#supplementary-data

Dataset 3.

Available for download at
https://journals.biologists.com/bio/article-lookup/doi/10.1242/bio.062345#supplementary-data

Biology Open • Supplementary information

Dataset 4.

Below are 40 female and 40 male fish. Some are young and some are old. Please score the fish on a scale from 0 to 5 on how frail you think the fish look. 0 is healthy, 5 is sickly frail and likely to die. If you feel the need, you can use half points.

When I think about frailty in this project, I think about what a frail human would look like.

Important note: we rarely/do not encounter extremely frail fish which I would score a 5, this is because such fish a removed from the aquarium beforehand, nevertheless you may have seen some in your career. Therefore, you should not feel obliged to use the entire range from 0-5, rather score them using your memory of "what is the frailest fish I have ever seen".

Females

Fish id	Image	Your score 0-5
253	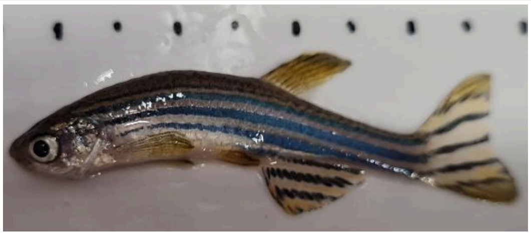	
15	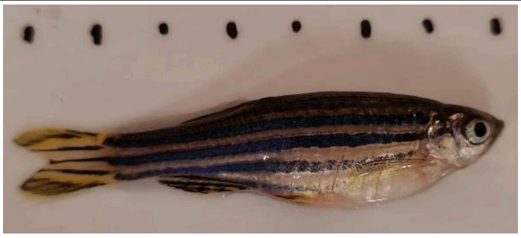	
258	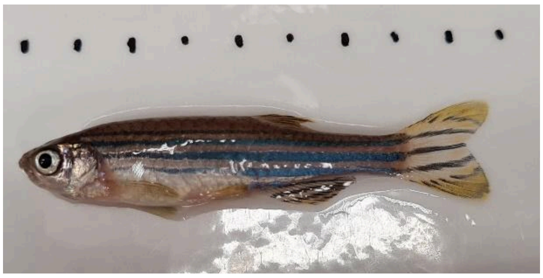	
241	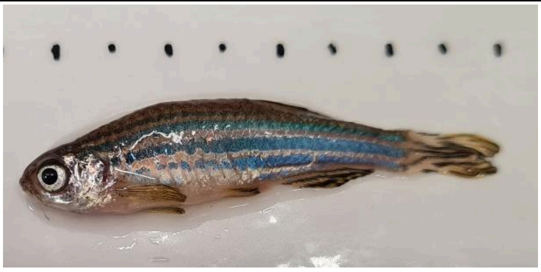	
16	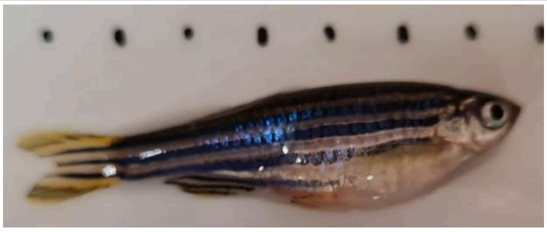	

Biology Open • Supplementary information

263	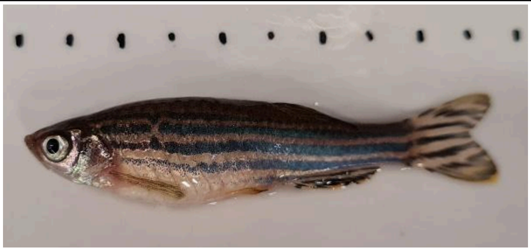	
245	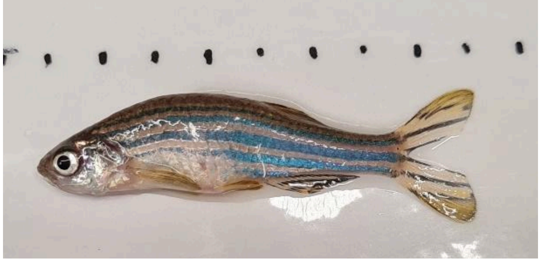	
6	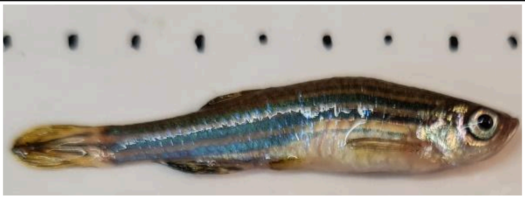	
278	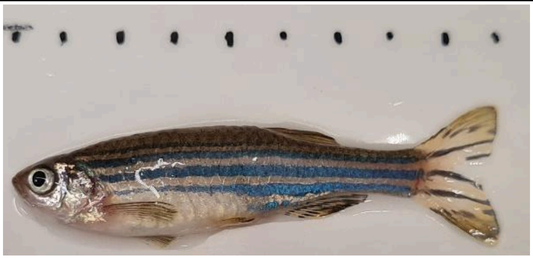	
279	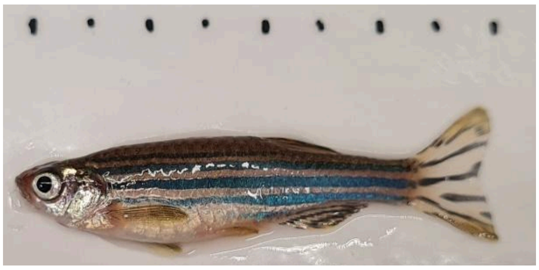	
303	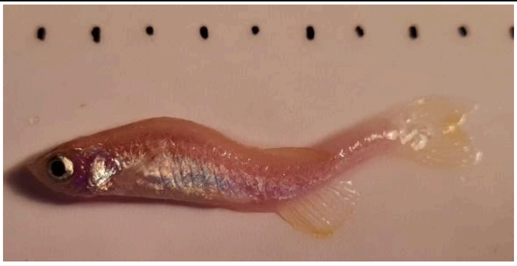	
17	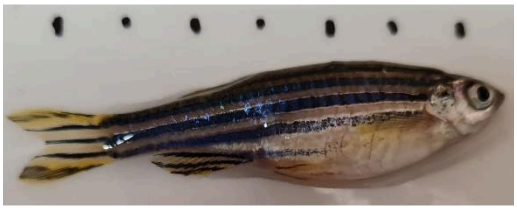	

Biology Open • Supplementary information

28	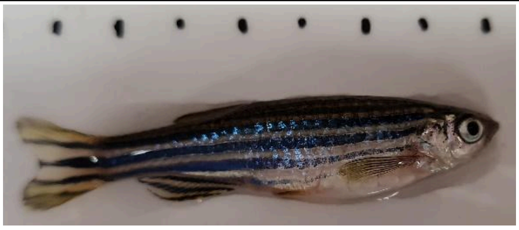	
305	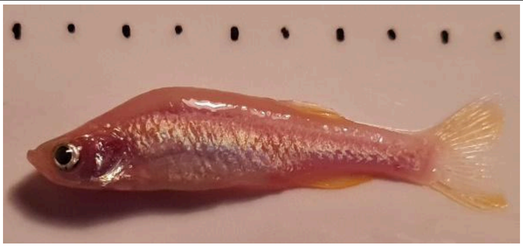	
246	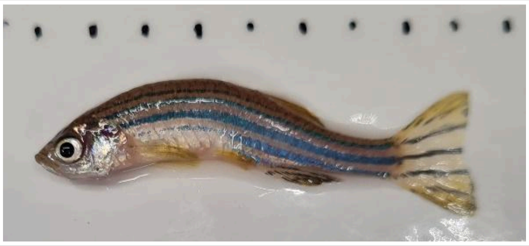	
292	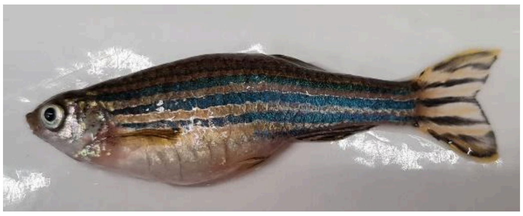	
309	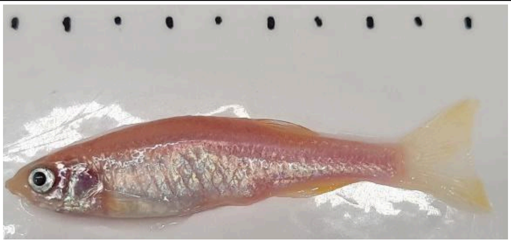	
336	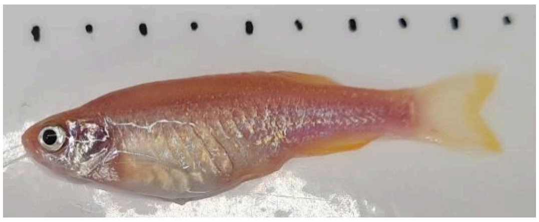	
264	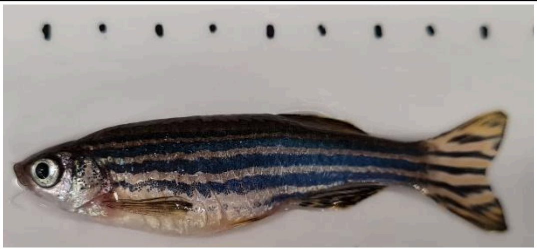	

29	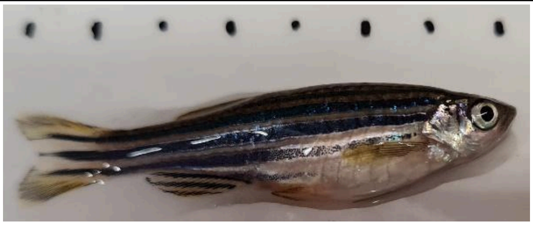	
30	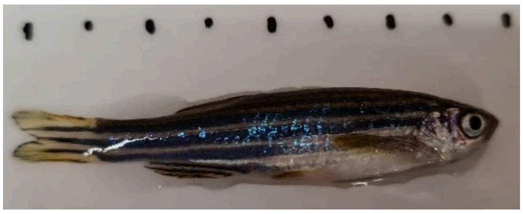	
251	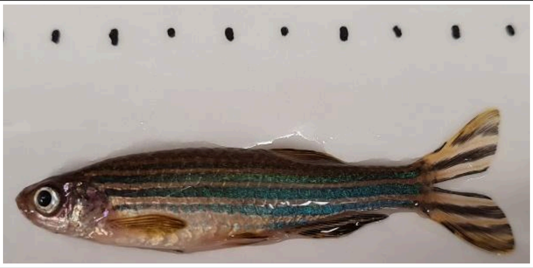	
275	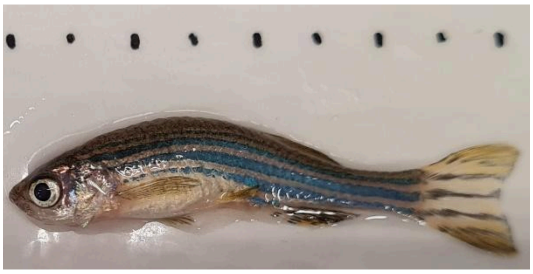	
42	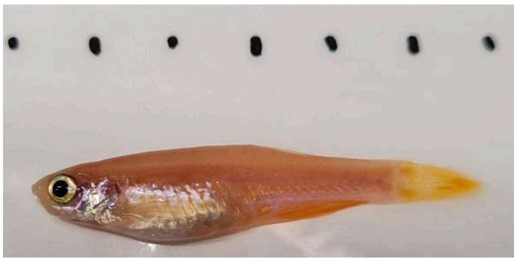	
277	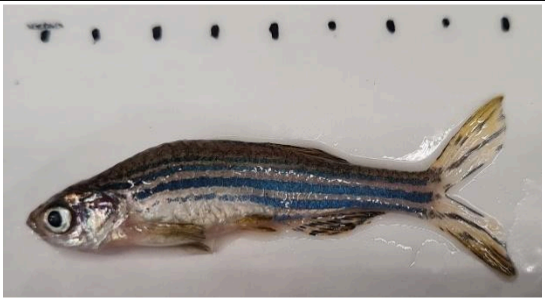	

Biology Open • Supplementary information

296	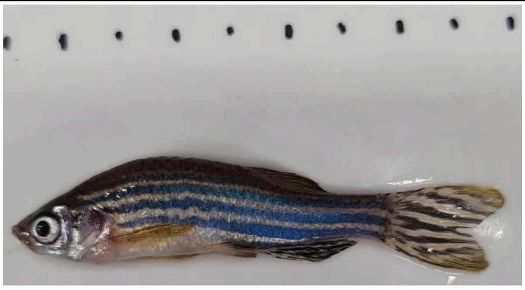	
302	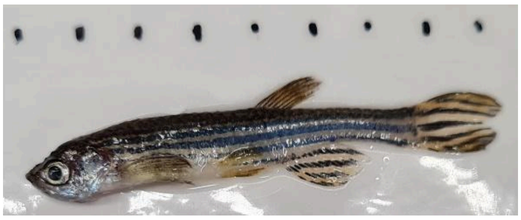	
44	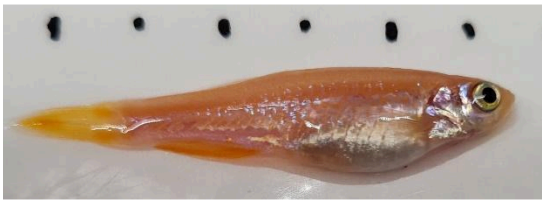	
304	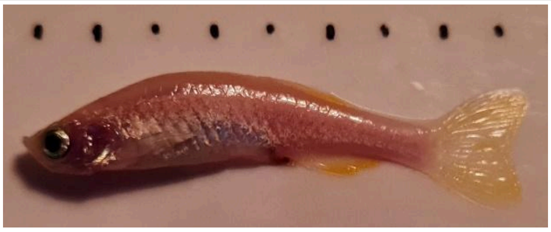	
301	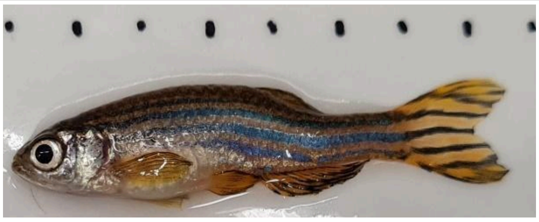	
300	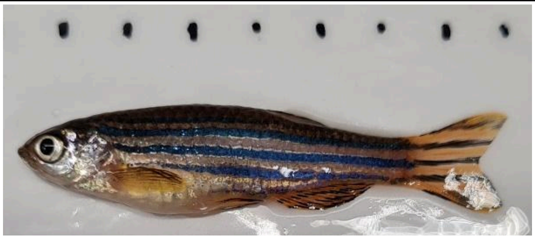	
289	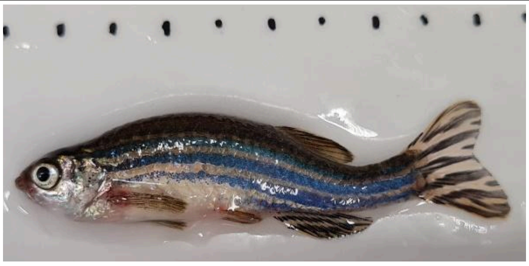	

337	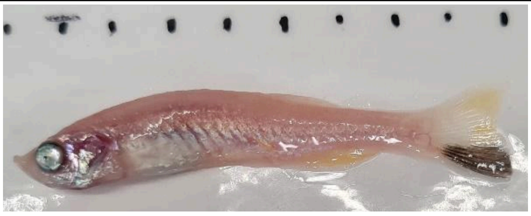	
131	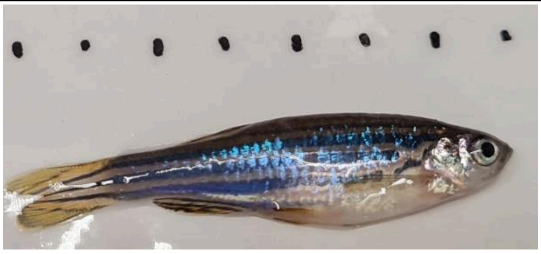	
306	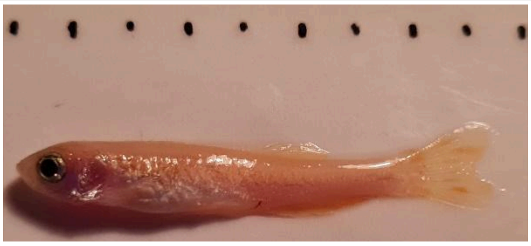	
307	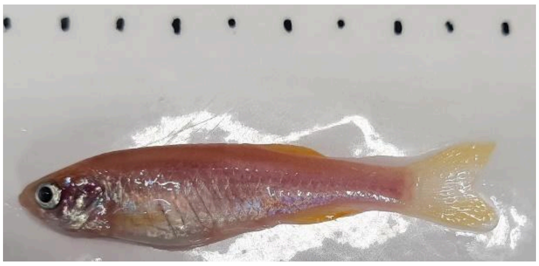	
290	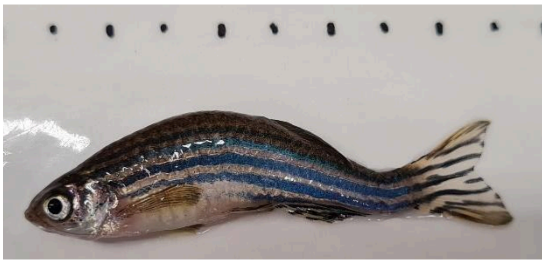	
308	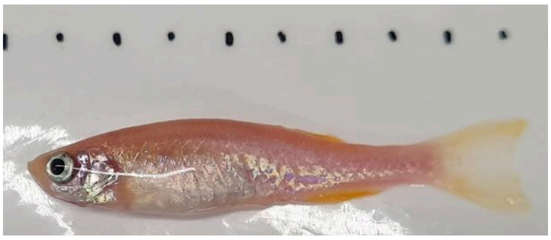	

Biology Open • Supplementary information

293	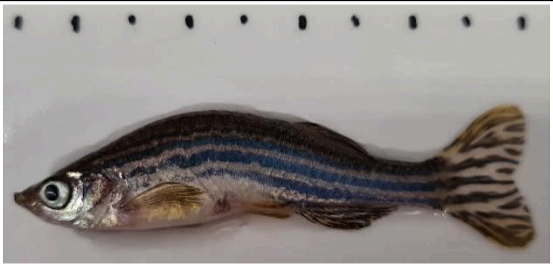	
291	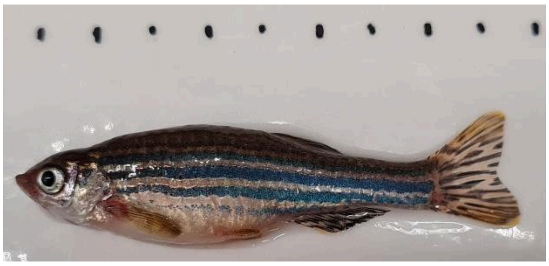	

Males

1	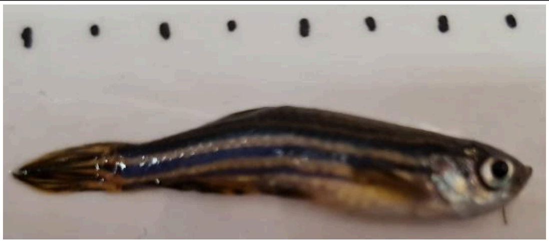	
202	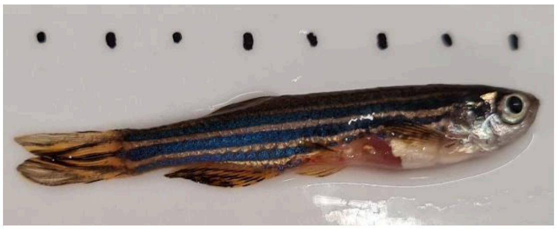	
11	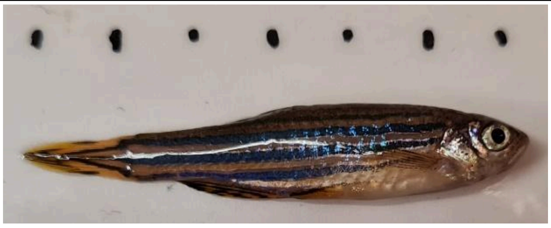	
199	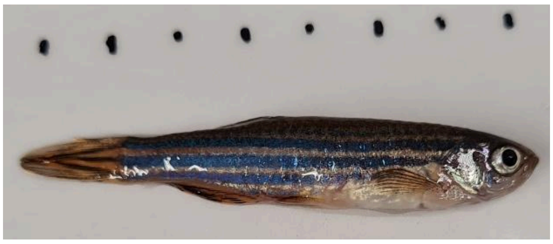	

Biology Open • Supplementary information

4	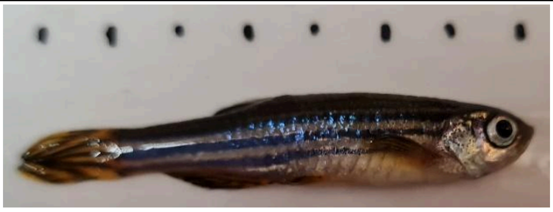	
38	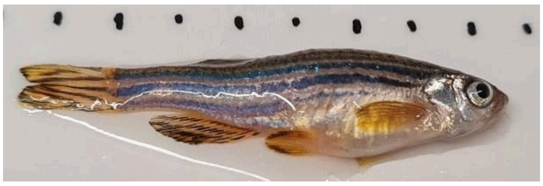	
12	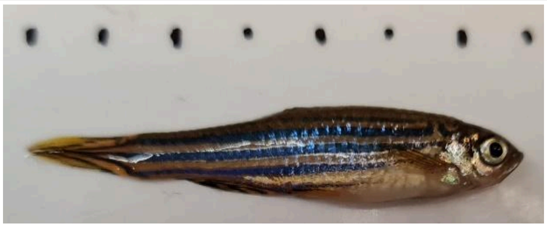	
272	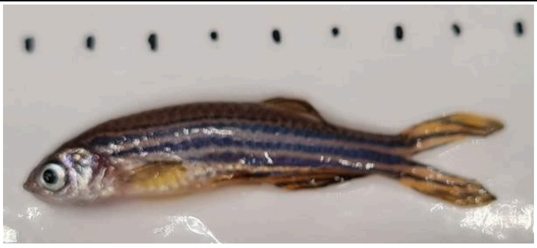	
25	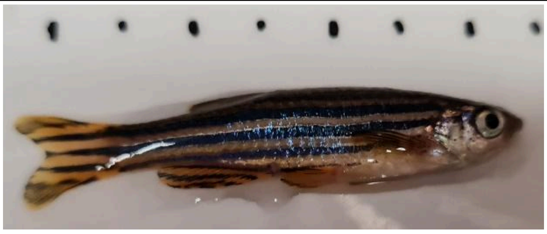	
3	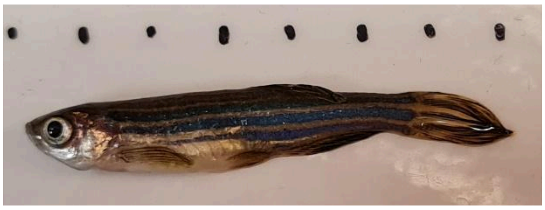	
37	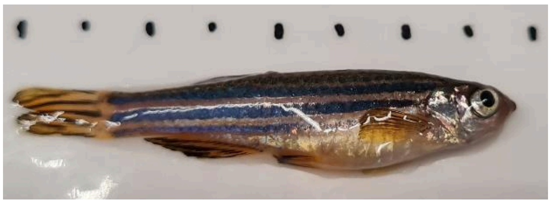	

198	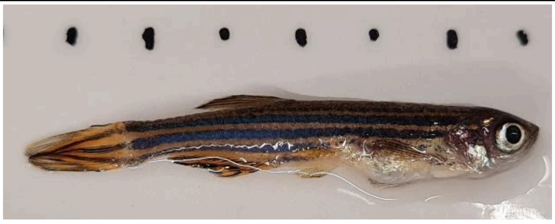	
49	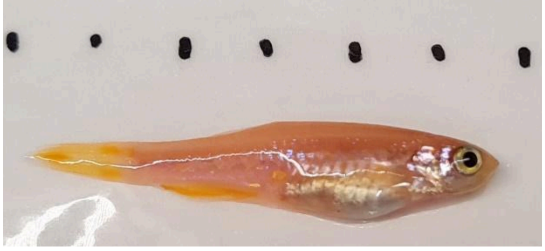	
123	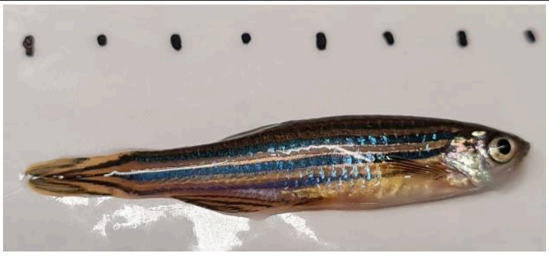	
343	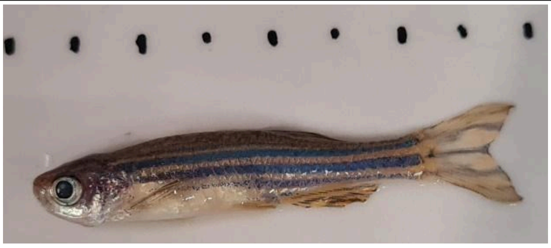	
205	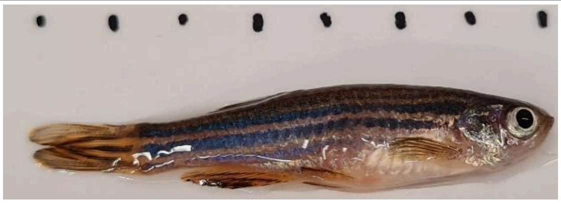	
102	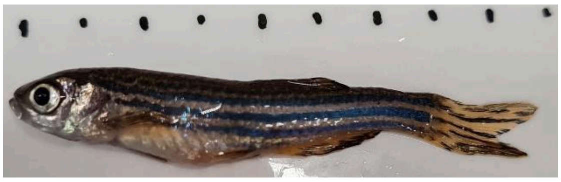	
2	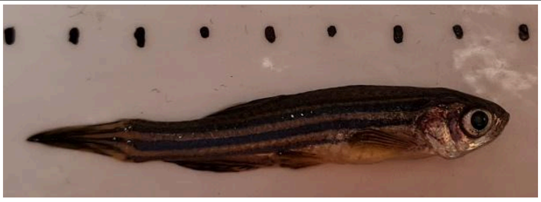	

Biology Open • Supplementary information

40	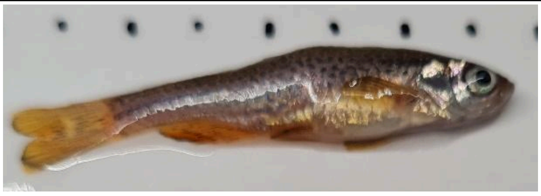	
13	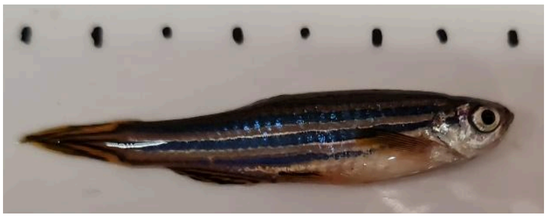	
206	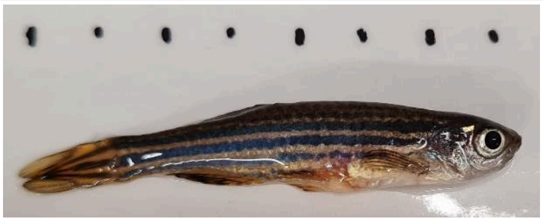	
41	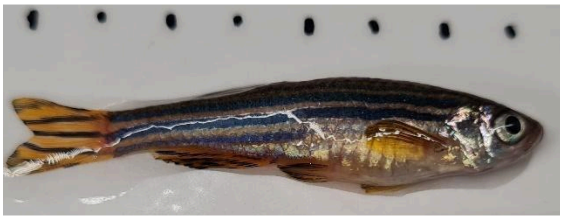	
103	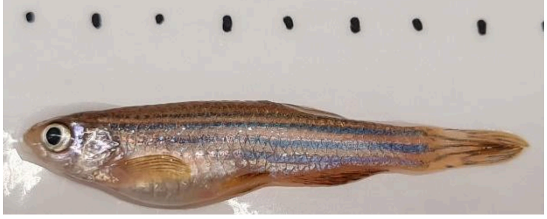	
47	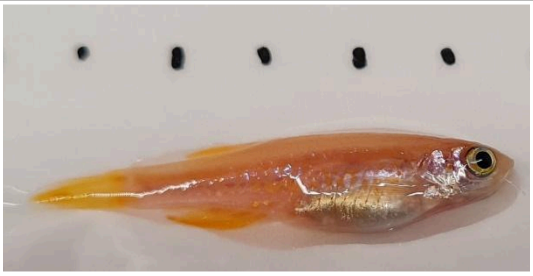	
344	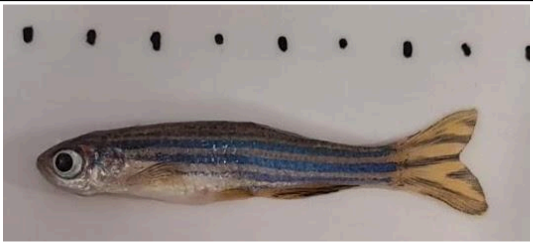	

26	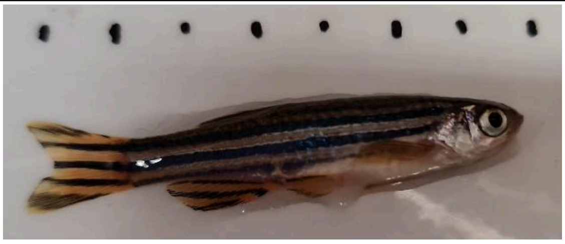	
338	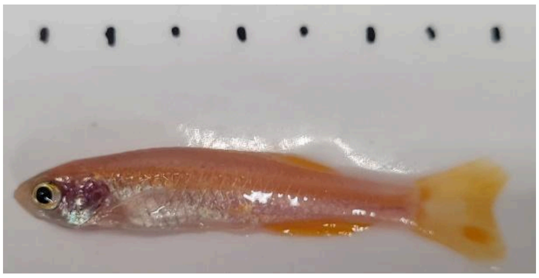	
339	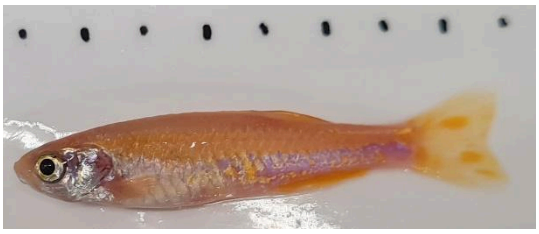	
348	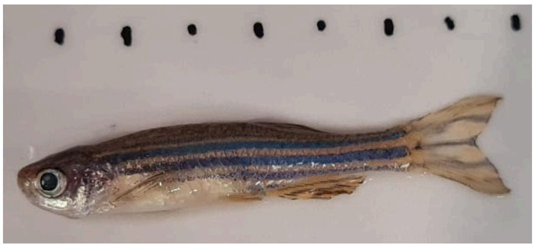	
97	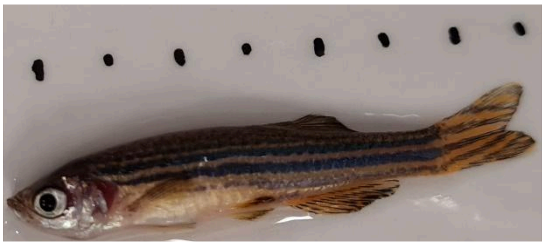	
48	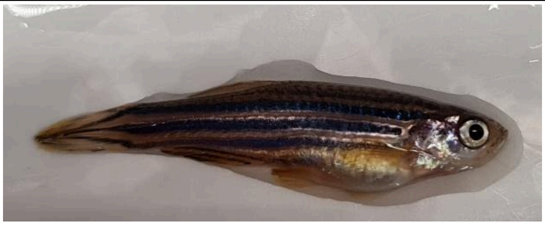	

204	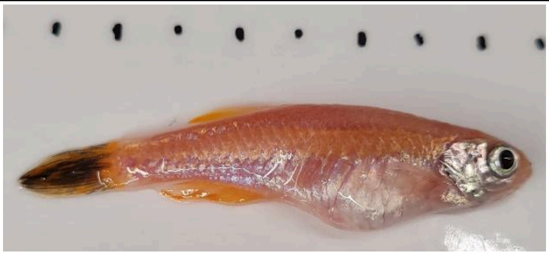	
197	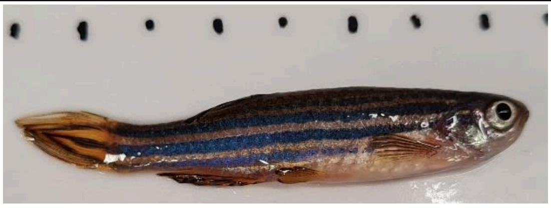	
340	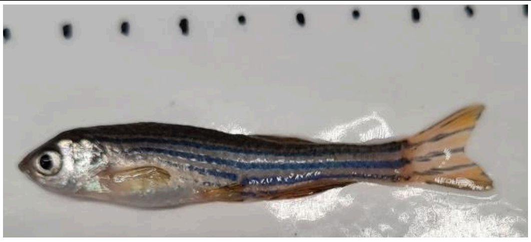	
349	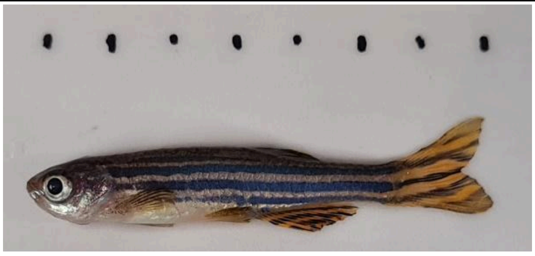	
350	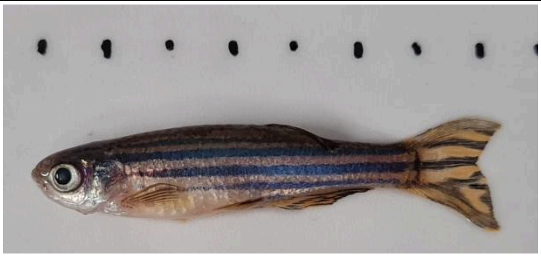	
98	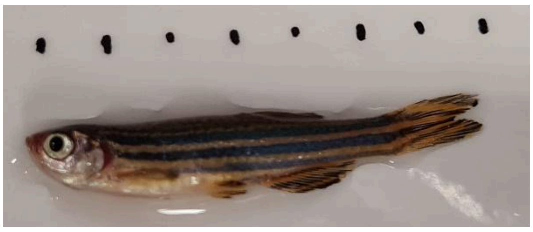	

Biology Open • Supplementary information

260	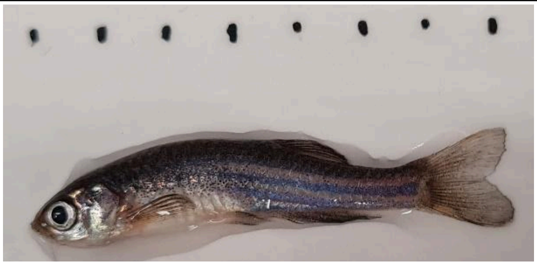	
112	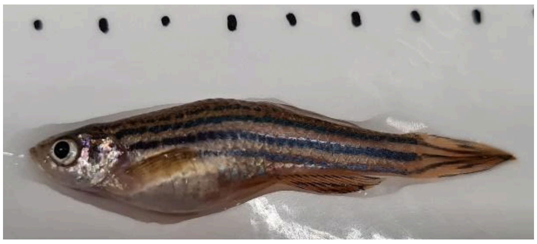	
27	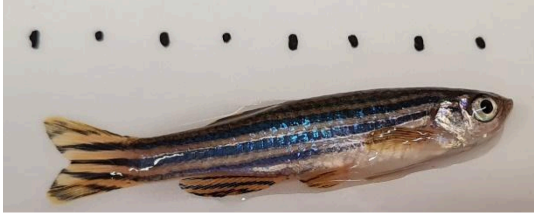	

Biology Open • Supplementary information

Dataset 5.

R script to create weights for the frailty index

```
# Import library
library(readxl)

# Import your data
data <- read_excel("C:/ _PATHWAY_.xlsx")

# Perform Linear Regression
regression_model <- lm(Expert ~ BMI + Spine + WL + Tumour + Fin, data = data)

# Summarize the model to see the coefficients
summary(regression_model)

# Extract the raw coefficients
raw_weights <- coef(regression_model)
print("Raw Coefficients (from regression):")
print(raw_weights)

# Extract only the criterion weights (excluding the intercept)
criterion_weights_raw <- raw_weights[names(raw_weights) != "(Intercept)"]

# --- Handle Negative Weights ---
# Set any negative weights to zero
positive_weights <- pmax(0, criterion_weights_raw)
print("Weights after setting negatives to zero:")
print(positive_weights)

# --- Normalize Weights to Sum to 1 ---
# This ensures your final optimized frailty index is also on a similar scale (e.g., 0-1)
optimized_weights <- positive_weights / sum(positive_weights)
```

Biology Open • Supplementary information

```
print("Optimized and Normalized Weights:")
print(optimized_weights)

# Verify they sum to 1
sum(optimized_weights)
```

Biology Open • Supplementary information
